# Supplementary material for: Latitudinal Variation in the Pattern of Temperature-Dependent Sex Determination in the Japanese Gecko, Gekko japonicus
Source: Animals (Basel). 2022 Apr 7;12(8):942. doi: 10.3390/ani12080942 (PMC9026794; doi:10.3390/ani12080942)
Supplement: Supplementary file 1 [file animals-12-00942-s001.zip › animals-1586666-supplementary.pdf]

Supplementary Figure

Figure S1 Air temperatures of the three location Yancheng (northern population), Chuzhou (central population) and Wenzhou (southern population). Data were extracted from the nearest climate station (< 8 km) for each location [Dataset of daily surface observation values in individual years (1981-2010) in China, China Meteorological Data Service Center, http://data.cma.cn/]. The dataset provided daily mean air temperatures, daily maximum air temperatures and daily minimum air temperatures of 30-year means. (a) mean air temperature; (b) maximum air temperature; (c) minimum air temperature.

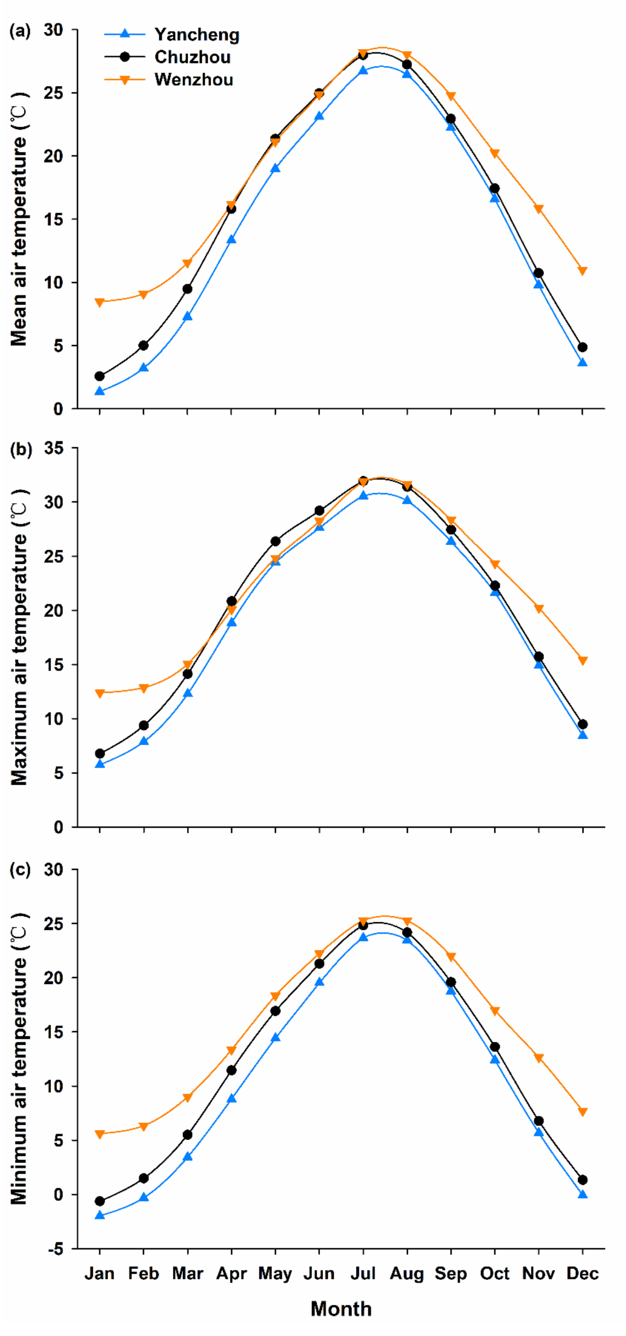

Supplementary Tables

Table S1 The number of hatchlings from each treatment dying at different ages (less than one month, one to two months, two to four months, and more than four months).

**Population**	**Incubation temperature/°C**	**Total hatchlings**	**< 1 month**	**1-2 months**	**2-4 months**	**>4 months**
Yancheng	24	59	3	8	30	18
	26	71	1	3	19	48
	28	61	4	3	4	50
	30	73	0	4	7	62
	32	49	0	6	7	36
Chuzhou	24	25	0	2	8	15
	26	40	2	2	4	32
	28	21	0	2	0	19
	30	29	3	1	4	21
	32	18	0	4	2	12
Wenzhou	24	52	3	2	7	40
	26	84	4	6	18	56
	28	49	2	1	6	40
	30	89	1	4	4	80
	32	70	2	3	16	49

Table S2 Corrected Akaike Information Criterion (AIC) values of Logistic and Flexit models for estimating the thermal reaction norm for sex ratio of *Gekko japonicus* from different populations, with Akaike weights in parentheses. Bolded values indicate the model that best fit the data based on AICc selection.

Population	Logistic	Flexit	Δ AICc
Yancheng	**32.00 (0.96)**	38.14 (0.04)	6.13
Chuzhou	**24.05 (0.99)**	32.94 (0.01)	8.88
Wenzhou	**34.22 (0.96)**	40.39 (0.04)	6.17
